# *Webervirus* phages exploit capsule- and O-antigen-targeting receptor-binding proteins to access diverse *Klebsiella pneumoniae* hosts

**DOI:** 10.1128/mbio.00046-26

**Published:** 2026-06-22

**Authors:** Zixuan Ding, Yongqi Mu, Wenyue Sun, Can Zhou, Yuqin Song, Shisong Jing, Dawei Wei, Jingnan Liang, Chao Wang, Haijian Zhou, Jie Feng

**Affiliations:** 1 Shandong First Medical University & Shandong Academy of Medical Sciences 518873, Jinan, China; 2 State Key Laboratory of Microbial Diversity and Innovative Utilization, Institute of Microbiology, Chinese Academy of Sciences 85387, Beijing, China; 3 College of Life Science and Technology, Key Laboratory of Protection and Utilization of Biological Resources in Tarim Basin of Xinjiang Production & Construction Corps, Tarim University 12483, Alar, China; 4 College of Life Science, University of Chinese Academy of Sciences 74519, Beijing, China; 5 Core Facility, Institute of Microbiology, Chinese Academy of Science, Beijing, China; 6 National Key Laboratory of Intelligent Tracking and Forecasting for Infectious Diseases, National Institute for Communicable Disease Control and Prevention, Chinese Center for Disease Control and Prevention 12415, Beijing, China; University of Pittsburgh, Pittsburgh, Pennsylvania, USA

**Keywords:** *Webervirus *phages, receptor-binding protein (RBP), lipopolysaccharide O-antigen, capsule

## Abstract

*Klebsiella pneumoniae* frequently downregulates or completely loses its capsule. This abolishes infection by capsule-targeting phages but concomitantly may favor infection by phages that exploit non-capsular receptors. Although capsule-targeting mechanisms have been studied in detail, the infection strategies of non-capsular phages remain poorly understood. Here, we analyzed receptor usage across a collection of *Webervirus* phages and found two contrasting phage-host interaction modes within *Webervirus*: one group relies on the capsular polysaccharide (CPS) to mediate productive infection, whereas infection by the other group is inhibited by the capsule, compellingly dependence on non-capsular receptors. Genome comparisons revealed a conserved morphogenesis backbone but highly variable receptor-binding protein (RBP) genes among *Webervirus* phages. These RBPs clustered into distinct clades that correlated with CPS-dependent and non-CPS receptor usage. Within the non-capsular clade, host genetics and complementation established the lipopolysaccharide O-antigen as the required receptor for these phages. Subsequent functional characterization of the RBPs confirmed that this clade specifically targets the O-antigen. Finally, exchanging RBPs by homologous recombination was sufficient to switch receptor usage, and combining phages with distinct receptor usage improved growth suppression in a representative clinical isolate. Together, these results define receptor-linked infection modes within *Webervirus* and show that RBP variation provides a modular route to diversify host range and increase robustness against capsule-state variability.

**IMPORTANCE:**

*Klebsiella pneumoniae* is a major multidrug-resistant pathogen, and its frequent capsule loss in clinical settings strongly affects phage therapy outcomes. Our work reveals two contrasting infection modes within *Webervirus* phages—capsule-dependent versus capsule-inhibited—and links each mode to distinct receptor-binding protein (RBP) clades. By identifying the O-antigen as a key non-capsular receptor, functionally validating O-antigen-targeting RBPs, and showing that RBP exchange can switch receptor usage and that combining complementary phages improves suppression of a clinical isolate, we provide a modular framework for rationally designing phage cocktails that remain effective despite capsule-state variability.

## INTRODUCTION

Bacteriophages (phages) are ubiquitous and among the most abundant and genetically diverse biological entities on Earth (1). By interacting with bacterial hosts, they shape microbial community structure and dynamics (2). A central feature of phage ecology is host range—the set of bacterial hosts a phage can infect—which reflects the specificity and flexibility of phage-bacterium interactions (3). Defining the mechanisms that govern host range is therefore essential for translating phage biology into predictable therapeutic activity (4).

*Klebsiella pneumoniae* is a clinically important gram-negative pathogen that causes hospital- and community-acquired infections and also colonizes the human gut, urinary tract, and respiratory tract, while persisting in diverse environmental niches (5, 6). Its polysaccharide capsule is a major virulence determinant and the primary receptor for most *Klebsiella* phages. Classical serotyping recognizes 77 capsular serotypes (K antigens), and more than 160 capsular loci (KL types) have been described (7, 8). This extensive capsule diversity strongly constrains phage specificity, such that many *Klebsiella* phages infect only one or two capsular types (9).

Capsule expression in *K. pneumonia*e is dynamic rather than fixed. We previously showed that some strains readily lose capsule during *in vitro* passage (10), and a longitudinal multi-center study of 1,017 clinical carbapenem-resistant *K. pneumoniae* isolates identified phenotypically capsule-deficient subpopulations, including 7.4% of KL64 and 12.2% of KL47 isolates (11). Although phages infecting capsule-deficient *K. pneumoniae* have been described, they remain less frequently reported than capsule-dependent phages (12, 13), limiting strategies to target populations that lose or remodel capsule.

Most *Klebsiella* phages initiate infection through receptor-binding proteins (RBPs) that specifically recognize surface structures on the bacterial cell, with the capsular polysaccharide (CPS) (K antigen) emerging as a dominant determinant of host tropism across many characterized *Klebsiella* phage-host interactions (9, 14). For capsule-targeting phages, RBPs couple CPS binding with depolymerase activity, and structural studies have defined the catalytic architecture and domain organization of depolymerase tailspikes, providing an important framework for interpreting structure predictions in newly identified RBPs (15–18). In contrast, *K. pneumoniae* also presents non-capsular receptors (e.g., lipopolysaccharide [LPS] core/core-OS, O-antigen, and outer membrane proteins), yet RBPs that target these features have been reported only sporadically. Examples include a K54 hypervirulent strain in which phage SCNJ1-Y uses a two-step mechanism with a capsule depolymerase for initial engagement and LPS as a secondary receptor for DNA ejection (19). Outer membrane proteins may also serve as secondary receptors. In particular, a conceptually similar two-step model was proposed earlier for the *Webervirus* phages NPat and Bmac, in which productive infection requires both specific CPS epitopes and the outer membrane porin OmpK36, supporting a capsule-first, outer-membrane-second mode of receptor engagement in *Klebsiella* phages (20). In addition, several studies point to RBP-mediated adsorption retargeting, in which mutations in tail-associated receptor-binding modules reshape receptor engagement to overcome capsule-mediated resistance. For example, in a K57 phage, point mutations in an auxiliary (“backup”) tail protein (ORF59) can redirect adsorption to an O-antigen polysaccharide site, thereby restoring infection of capsule-resistant hosts (21). In the minimalist phage MMNM, genetic and evolutionary analyses implicate the LPS O1 antigen as the required receptor, with recurrent substitutions in tail/baseplate proteins consistent with adaptive tuning of O-antigen binding (22). Nevertheless, non-capsular RBP usage has not been systematically examined, leaving gaps in how phages access capsule-deficient or capsule-modified *K. pneumoniae*.

Here, we show that *Webervirus* phages adopt two distinct infection strategies against *K. pneumoniae*. One group follows a “capsule-first” route mediated by CPS-targeting RBPs, whereas the other infects capsule-deficient strains with a broader pattern that depends on O-antigen recognition. *Webervirus* RBP phylogeny reveals a clear split between O-antigen- and capsule-targeting types that matches their functional properties. Moreover, swapping RBPs between *Webervirus* phages is sufficient to reprogram host preference, switching recognition from O-antigen to capsule. Together, these findings show how capsule- and O-antigen-targeting RBPs enable *Webervirus* phages to access diverse hosts and highlight modular evolution of host-recognition machinery as a key driver of adaptation to variable bacterial surface architectures.

## RESULTS

### Modular infection of encapsulated *K. pneumoniae* and broad infection of capsule-deficient derivatives

Analysis of the NCBI Virus database indicated that *Webervirus* is one of the most abundant genera among *Klebsiella* phages (10.2%, Fig. S1). We therefore focused on *Webervirus* and selected 47 *Webervirus* phages from our collection for further study (Table S1). To evaluate their host range, we assembled a diverse panel of 51 clinical *K. pneumoniae* isolates encompassing broad coverage of capsule and O-antigen diversity, including 22 capsule (KL) types and 6 O-antigen types (Fig. S2). All isolates harbored acquired antimicrobial resistance genes conferring resistance to at least three classes of antibiotics, and 58.8% (30/51) carried *bla*_KPC-2_ and/or *bla*_NDM-1_ genes.

**Fig 1:**
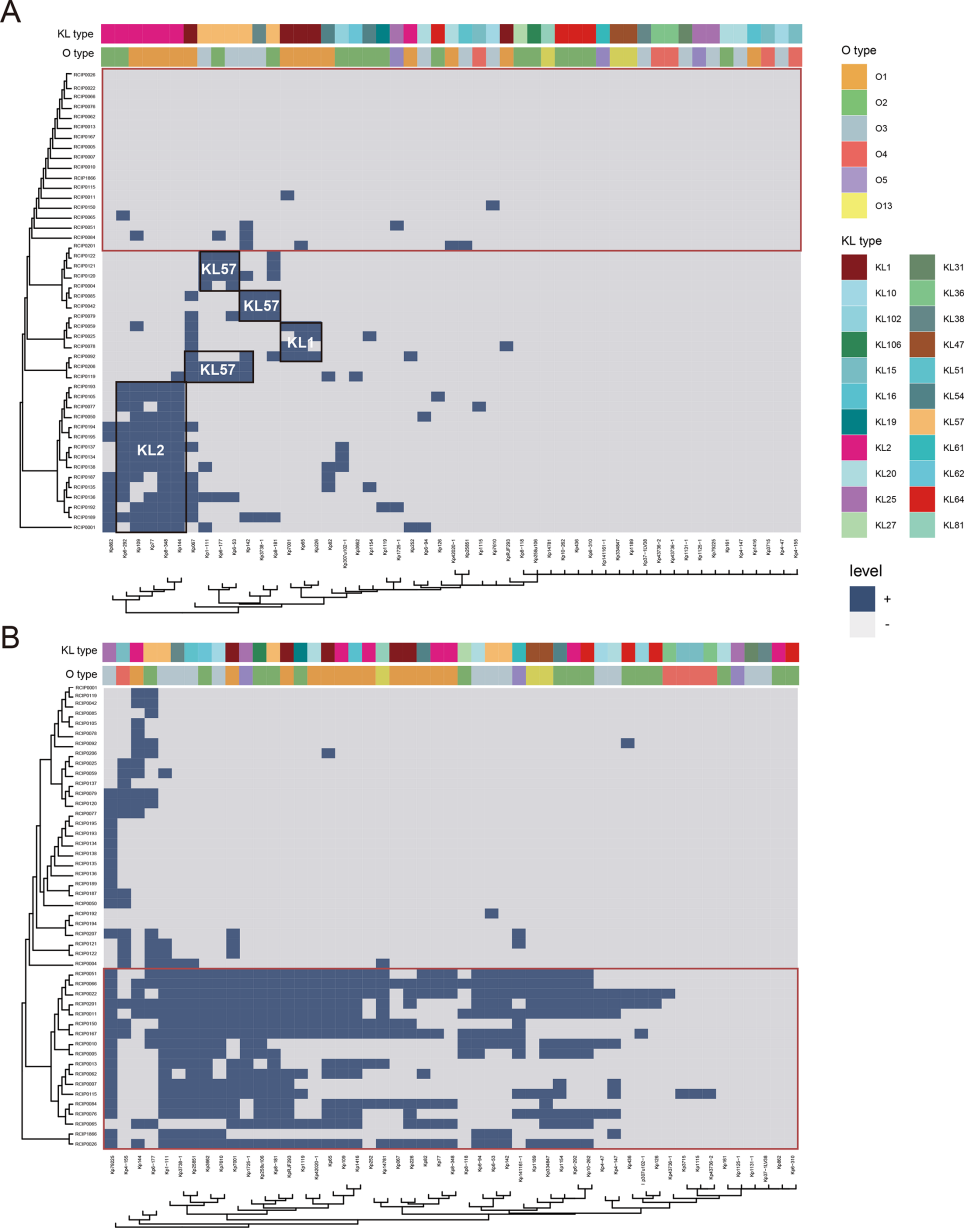
Lysis spectra of *Webervirus* phages against encapsulated and capsule-deficient *K. pneumoniae* hosts. (**A**) Phage-bacterium incidence matrix generated by challenging 51 encapsulated (wild-type) *K. pneumoniae* strains with 47 *Webervirus* phages. Phages are arranged in rows and hosts in columns; both axes are hierarchically clustered. Blue squares indicate lysis, evidenced by visible plaque formation. Colored strips above the matrix denote capsular locus (KL) types and O-antigen types. (**B**) Phage-bacterium incidence matrix generated by challenging the matched capsule-deficient derivatives of these 51 *K. pneumoniae* strains with the same 47 *Webervirus* phages. Phages are arranged in rows and hosts in columns; both axes are hierarchically clustered. Blue squares indicate lysis, evidenced by visible plaque formation. Colored strips above the matrix denote capsular locus (KL) types and O-antigen types.

We evaluated the host ranges of 47 *Webervirus* phages against 51 *K. pneumoniae* isolates by spot assay, generating a comprehensive phage-host matrix of 2,397 pairings (Fig. 1A). Hierarchical clustering of the bi-adjacency matrix revealed distinct phage-host interaction patterns. These patterns could be categorized into two groups based on host coverage. The first group comprised 18 phages that exhibited no lytic activity or lysed only a limited number of strains. The second group consisted of 29 phages displaying high KL-specificity, forming distinct infection modules corresponding to KL1, KL2, and KL57.

To understand the ability of these phages to lyse capsule-deficient *K. pneumoniae*, we conducted three to five serial *in vitro* passages of the 51 isolates (10). The resulting variants formed translucent colonies, which served as indicators of capsule deficiency (Fig. S2A). PCR fragment sequencing of the *cps* (capsule biosynthesis) gene cluster revealed that representative variants harbored insertion sequence (IS) insertions or mutations. Transmission electron microscopy (TEM) of strain Kp8-181 variant confirmed the complete loss of the capsular layer (Fig. S3B). Uronic acid-based quantification of capsular polysaccharide across four strain backgrounds (KpRJF293, Kp8-181, Kp4-147, and Kp1189) showed significantly higher uronic acid levels in encapsulated strains than in their corresponding capsule-deficient derivatives, indicating a marked reduction of CPS in the capsule-deficient variants (Fig. S3C).

We then analyzed phage interactions with this capsule-deficient panel and observed a dramatic shift in the lysis patterns compared to their encapsulated counterparts. Hierarchical clustering of the bi-adjacency matrix revealed two distinct phage groups. One cluster comprised 18 phages that had previously shown no or only limited lytic activity against encapsulated strains, but displayed broad host ranges across the capsule-deficient panel, with substantially expanded lysis profiles (Fig. 1B). Conversely, the other 29 phages, which had shown KL-specific lysis of encapsulated strains, exhibited no or minimal lytic activity against capsule-deficient strains. These results indicated that *Webervirus* phages included two groups, with one phage group being capsule-inhibited and the other being capsule-dependent for successful infection.

### Phylogeny of *Webervirus* RBPs reveals a putative division between capsular and non-capsular receptor-targeting types

**Fig 2:**
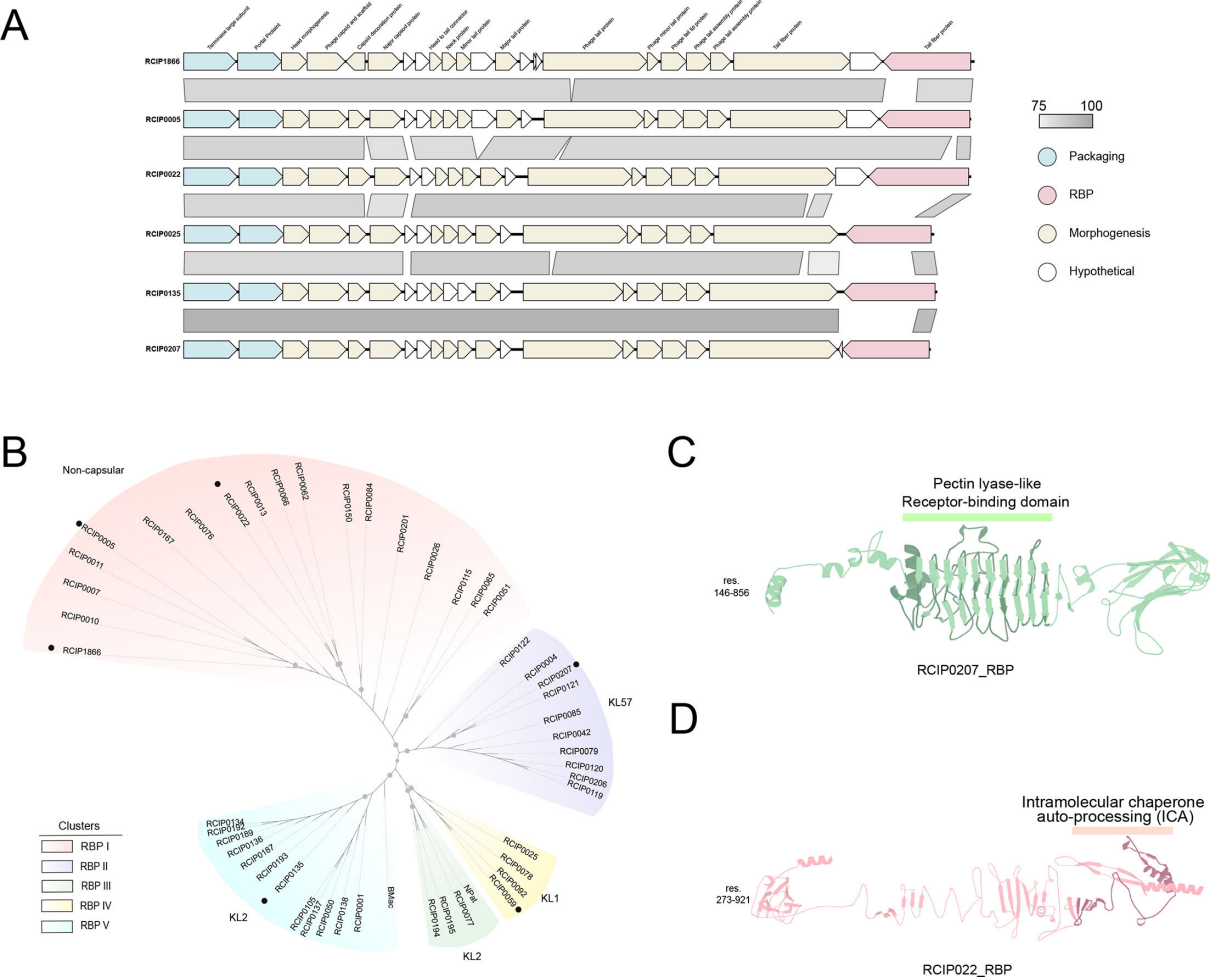
*Webervirus* RBPs fall into two distinct types. (**A**) Comparative genomic analysis of the morphogenesis module in representative *Webervirus* phages. Arrows represent coding sequences and are colored by predicted function: blue, packaging; yellow, morphogenesis; pink, RBPs; gray, general functions; and white, hypothetical proteins. Gray shading denotes regions >70% nucleotide identity. (**B**) Maximum-likelihood phylogeny of *Webervirus* RBP amino-acid sequences, including the reference RBPs from BMac and NPat. Major clusters are labelled RBP I–V. Bootstrap support values exceeding 90% are shown at the corresponding nodes; black-filled circles indicate RBPs that were functionally characterized in this study. (**C**) Predicted structure of the representative lyase-like receptor-binding protein RCIP0207_RBP. The pectate lyase-like receptor-binding domain is indicated above the model and highlighted in the structure. (**D**) Predicted structure of a representative non-depolymerase RBP (RCIP0022_RBP). The intramolecular chaperone auto-processing (ICA) region is indicated and highlighted.

Phages recognize their hosts through RBPs, which assemble into fibers or spikes at the distal tail (23). Because the infection patterns described above segregated into two distinct patterns, we hypothesized that *Webervirus* phages encode RBPs targeting either CPS or non-CPS receptors. Comparative genomics of *Webervirus* phages revealed that morphogenesis gene clusters share >70% nucleotide identity, with a notable exception at the putative RBP gene (Fig. 2A). To clarify the basis of these two infection patterns, a phylogenetic tree constructed from the 47 putative RBPs identified in this study, together with the reference RBPs from BMac and NPat, resolved into five clades, designated clusters I–V, which mapped closely to receptor usage and host-range phenotype (Fig. 2B).

These clades reflected distinct host-range patterns. Cluster I comprised RBPs from phages whose infection was blocked by capsule and was mainly observed on capsule-deficient derivatives, indicating recognition of non-CPS receptors. By contrast, clusters II–V contained RBPs from phages with KL-specific host ranges. Cluster II was associated with phages targeting KL57 strains whereas cluster IV corresponded to KL1-targeting phages. RBPs from KL2-targeting phages were separated into two distinct clades, clusters III and V. Notably, the previously characterized KL2-targeting *Webervirus* phages BMac and NPat grouped within one of these KL2-associated clades, providing additional support for the linkage between this lineage and KL2 recognition. Together, these results indicated that RBPs associated with the same capsular specificity can remain phylogenetically separated, suggesting diversification of capsule-targeting RBPs despite convergence in host-range phenotype.

Notably, cluster I RBPs lack recognizable depolymerase-associated signatures, whereas RBPs in clusters II–V are annotated with lyase-like domains or, when annotation is ambiguous, exhibit predicted β-strand-rich folds typical of polysaccharide depolymerases (Table S3, Fig. S4). To further investigate structural differences among these RBPs, we compared the predicted structures of representative RBPs from different clusters using AlphaFold3. After trimming predicted disordered regions, the cluster II RBP RCIP0207_RBP showed an overall architecture similar to that of the cluster V RBP BMac_RBP and contained a prominent pectate lyase-like receptor-binding domain, consistent with a depolymerase-type structure. In contrast, the cluster I RBP RCIP0022_RBP lacked a lyase-like domain and was instead predicted to contain an intramolecular chaperone auto-processing (ICA) domain (Fig. 2C and D). Consistent with this prediction, recombinant cluster I RBPs, including RCIP0022_RBP and the corresponding GFP fusions, migrated predominantly as a lower-molecular-weight product on SDS-PAGE, with reduced full-length band intensity, suggesting ICA domain-mediated self-cleavage during maturation (Fig. S5).

We next functionally tested CPS-directed activity by expressing and purifying representative RBPs from clusters II, IV, and V, and assessed their activity using spot tests on bacterial lawns of strains carrying the KL57, KL1, and KL2 capsule types. All three RBPs generated semi-clear spots on lawns of the corresponding KL strains but not on those of the matched CPS-deficient derivatives (Fig. S6A). To further validate this activity, CPS was extracted from each strain and subjected to *in vitro* digestion with the corresponding RBPs. SDS-PAGE analysis revealed marked CPS degradation upon RBP treatment, consistent with CPS depolymerase activity (Fig. S6B).

### Cluster I *Webervirus* phages exploit O-antigen to infect capsule-deficient *K. pneumoniae*

To identify the non-capsular receptor targeted by these phages, we used RCIP0022—which infects capsule-deficient *K. pneumoniae*—and KpRJF293cap^-^ as a model pair. Serial passage yielded RCIP0022-resistant KpRJF293cap^-^ mutants, and whole-genome sequencing of three independent clones revealed recurrent disruptive mutations in O-antigen biosynthesis genes (e.g., IS insertions and premature stop codons; Fig. S7A), implicating the O-antigen as the receptor for RCIP0022.

We next asked whether O-antigen-mediated infection represents a broadly relevant route in capsule-deficient *K. pneumoniae*. Analysis of 2,756 complete *K. pneumoniae* genomes in NCBI RefSeq (March 2025) showed that four O types account for >90% of genomes: O2 (37.8%), O1 (32.7%), O3 (12.4%), and O13 (7.9%) (Fig. S7B). O1 is structurally related to O2 and depends on O2-locus functions plus O1-specific *wbbY/Z*, whereas O3 is a polymannose O-antigen and O13 specifies a distinct disaccharide repeat with a terminal cap (24) (Fig. S7C).

Guided by this distribution, we assembled a capsule-deficient strain panel representing these four O types (O1/KpRJF293, O2/Kp8-181, O3/Kp4-147, and O13/Kp1189) and generated matched O-antigen-deficient derivatives by deleting key biosynthesis genes (Δ*wbbN* in O1 and O2, Δ*wbdD* in O3, and Δ*wbbB* in O13) to abolish O-antigen production. LPS silver staining confirmed loss of high-molecular-weight O-antigen bands in all mutants (Fig. S8).

**Fig3:**
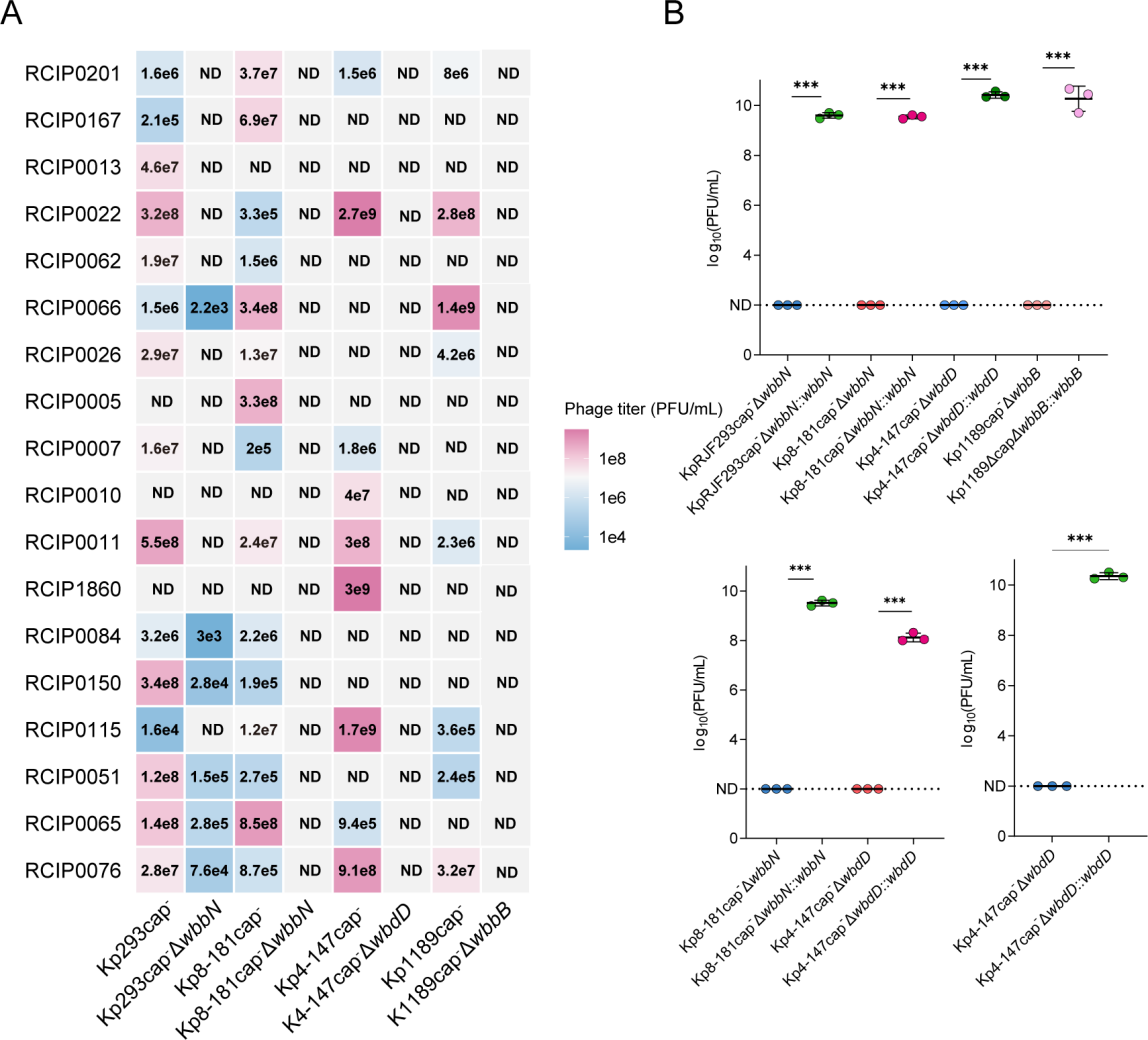
The O-antigen serves as a critical receptor for capsule-inhibited *Webervirus* phages to infect. (**A**) Interaction matrix of 18 *Webervirus* phages across four *K. pneumoniae* strains tested in a capsule-deficient background (cap^−^) and in the corresponding O-antigen-deficient derivatives (KpRJF293cap^−^Δ*wbbN*, Kp8-181cap^−^Δ*wbbN*, Kp4-147cap^−^Δ*wbdD*, or Kp1189cap^−^Δ*wbbB*, as indicated). Each cell shows the phage titer measured by plaque assay, expressed as PFU/mL. ND indicates titers below the limit of detection (<100 PFU/mL). Color shading represents phage titer on a log-scaled gradient. (**B**) Phage titers measured by plaque assay on O-antigen-deficient *K. pneumoniae* mutants and their corresponding complemented strains for the representative capsule-inhibited phages RCIP0022, RCIP0005 and RCIP1866. Points represent independent measurements, and horizontal bars indicate the mean. ND denotes titers below the limit of detection (<100 PFU/mL), indicated by the dotted line. ***P < 0.001 (statistical test specified in Materials and Methods).

We then evaluated the host ranges of 18 phages against the four capsule-deficient hosts and their O-antigen-deficient derivatives. In the capsule-deficient backgrounds, phage infectivity was detected across a range of O-antigen types, but the breadth varied among phages (Fig. 3A). Of the 18 phages, 4/18 (22.2%) were restricted to a single O type (two O3-only and two O2-only), whereas 14/18 (77.8%) produced detectable lysis across two or more O types. Specifically, 5/18 (27.8%) lysed two O types (all with an O1 or O2 profile), 4/18 (22.2%) lysed three O types, and 5/18 (27.8%) lysed all four O types, indicating a continuum of O-type lysis breadth.

When the corresponding O-antigen-deficient derivatives were tested, titers were markedly reduced for most phage-host combinations relative to the matched capsule-deficient parental strains, and many fell to the limit of detection (Fig. 3A). Using the matched capsule-deficient parental strains as reference hosts, efficiency of plating (EOP) analysis showed that O-antigen deficiency caused a pronounced loss of plating efficiency in most cases. In most phage-host combinations, plaque formation was no longer detectable, whereas a subset of combinations retained measurable but reduced plaque formation, with the extent of reduction differing among phages and host backgrounds (Table S4).

Representative phages showed the same overall pattern. RCIP1866, RCIP0005, and RCIP0022 produced high titers on O-intact hosts, whereas titers on the matched O-antigen-deficient derivatives were strongly reduced or fell to the limit of detection. Following complementation of the deleted O-antigen biosynthesis locus, plaque formation was restored, and phage titers increased markedly (Fig. 3B). Overall, variation in host-range breadth under capsule-deficient conditions primarily reflected differences in O-type coverage rather than KL diversity.

### Functional characterization of O-antigen RBPs

Having established O-antigen as the key receptor for infection in capsule-deficient conditions, we next sought to identify the viral determinant that mediates O-antigen recognition. Bioinformatic annotation suggested that cluster I O-antigen-targeting RBPs lack recognizable polysaccharide depolymerase domains found in clusters II–V, consistent with an absence of enzymatic O-antigen (OPS)-degrading activity. We therefore hypothesized that cluster I RBPs may instead bind the O-antigen *via* a non-enzymatic distal receptor-binding domain, analogous to the phage T5 lateral tail fiber pb1 (25, 26).

**Fig 4:**
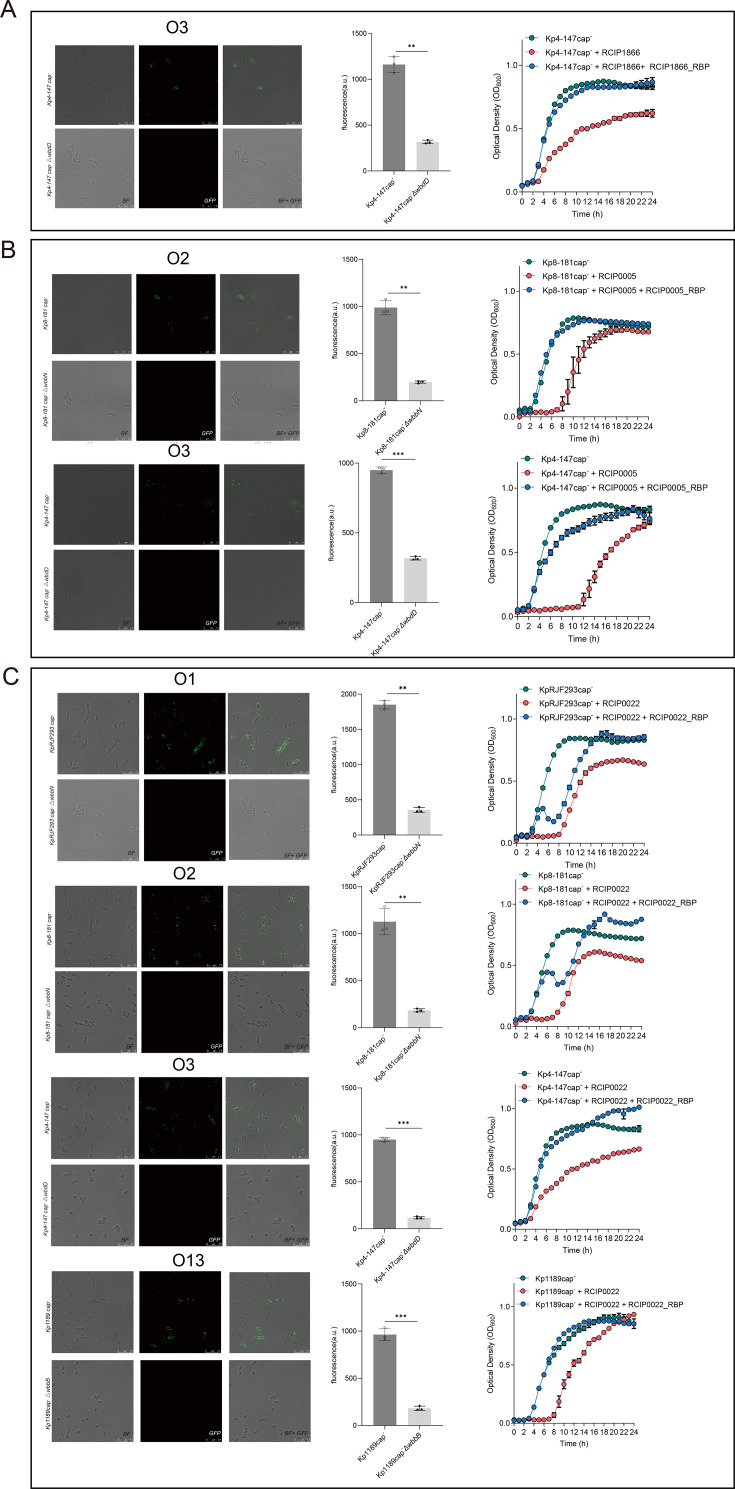
Functional characterization of RBPs represented by cluster I. (**A**) Binding and competition activity of RCIP1866_RBP on the O3 capsule-deficient host Kp4-147 and its matched O-antigen-deficient derivative. Left, fluorescence microscopy of GFP-tagged RBP binding; middle, fluorometric quantification of cell-associated GFP signal; right, growth curves showing phage infection in the absence or presence of purified unlabeled RBP. (**B**) Binding and competition activity of RCIP0005_RBP on O2 and O3 capsule-deficient hosts and their matched O-antigen-deficient derivatives. (**C**) Binding and competition activity of RCIP0022_RBP on O1, O2, O3, and O13 capsule-deficient hosts and their matched O-antigen-deficient derivatives. Statistical significance is indicated as **P < 0.01 and ***P < 0.001 (statistical test specified in Materials and Methods).

To test whether cluster I RBPs mediate O-antigen recognition, we selected three representative RBPs spanning distinct O-type breadths and assessed their interactions with strains expressing different O-antigen variants. RBPs were expressed as GFP fusions and evaluated for binding to capsule-deficient strains and matched derivatives lacking both capsule and O-antigen. For RCIP1866_RBP, confocal microscopy revealed clear surface-associated GFP signals on Kp4-147 cap^-^, whereas this binding signal was lost in the matched O-antigen-deficient derivative (Fig. 4A). Consistently, fluorescence spectrophotometric measurements confirmed robust binding of RCIP1866_RBP to the O3 antigen of Kp4-147. To further substantiate the functional relevance of RBP binding, we designed a competition assay. Preincubation of Kp4-147 cap^-^ with RCIP1866_RBP reduced adsorption of RCIP1866 and consequently prevented phage-mediated lysis, supporting a competitive occupancy model for O-antigen binding sites. Extending these readouts to additional cluster I RBPs revealed distinct O-type recognition breadths: RCIP0005_RBP recognized O2 and O3 (Fig. 4B), whereas RCIP0022_RBP displayed the broadest profile, recognizing O1, O2, O3, and O13 (Fig. 4C). Together, these results indicate that differences in O-antigen tropism among these phages are attributable to their distinct RBPs, with each RBP conferring a characteristic O-type recognition spectrum that defines the host range of its cognate phage under capsule-deficient background.

### Host tropism rewiring and resistance constraints driven by RBP exchange

Comparative genomics across *Webervirus* suggests a simple but flexible principle: each genome encodes a single RBP at a conserved locus, while RBP sequences diversify into discrete clusters that broadly correlate with O-antigen- versus capsule-associated receptor usage (Fig. S9A). In contrast, a terminase large subunit (TerL)-based phylogeny of the same 47 phages shows CPS- and O-antigen-associated RBPs intermingled across the backbone tree, indicating that receptor preference is not strictly predicted by overall relatedness (Fig. S9B). Together, these patterns are consistent with RBP changes—potentially including horizontal exchange—driving host-range diversification on an otherwise conserved genomic backbone, in line with modular RBP architectures described in other tailed phages (27).

**Fig 5:**
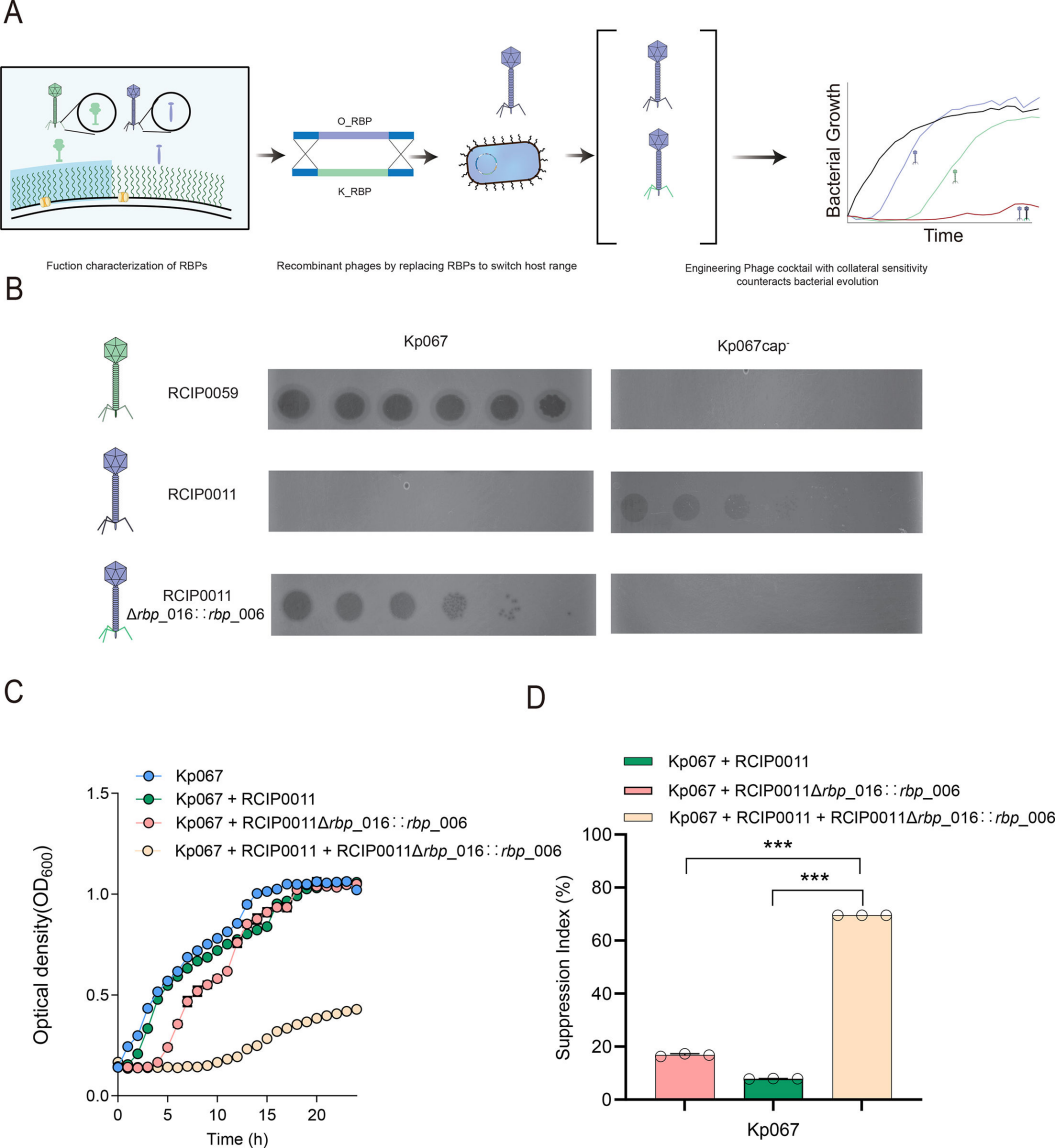
Swapping RBP switches receptor usage. (**A**) Engineering rationale and workflow. (**B**) Spot-assay validation host-range shifts: the recombinant phage RCIP0011Δ*rbp*_016∷*rbp*_006 lost lytic activity on its original O-antigen host but acquired infectivity toward the K1-encapsulated strain. (**C**) Growth curves of *K. pneumoniae* Kp067 challenged with RCIP0011, RCIP0011Δ*rbp*_016∷*rbp*_006, or a two-phage cocktail containing both phages. OD_600_ was recorded for 24 h. Curves show the mean of three independent experiments. (**D**) Growth suppression at 24 h relative to untreated Kp067, calculated as described in Materials and Methods. Bars show means, with individual points representing independent experiments. ***P < 0.001; statistical tests are described in Materials and Methods.

We therefore hypothesized that O-antigen and CPS tropism could be interconverted by RBP exchange. Using RCIP0011 (an O-antigen-targeting phage with broad O-type coverage across the four dominant O types) as the recipient, we replaced its native RBP with the K1 capsule-targeting RBP from RCIP0059 *via* homologous recombination, generating RCIP0011Δ*rbp*_016∷*rbp*_006 (Fig. 5A). The recombinant gained robust lytic activity against K1 strains and lost lysis of the original O1 host (Fig. 5B), demonstrating that swapping a single RBP can reprogram receptor usage. In parallel, we observed collateral sensitivity: CPS loss conferred resistance to CPS-targeting phages but increased susceptibility to O-antigen-targeting phages, and combining both phage types reduced resistance emergence relative to single-phage treatment (28).

In a representative clinical strain (Kp067), growth curves showed minimal suppression by RCIP0011 alone, transient suppression with RCIP0011Δ*rbp*_016∷*rbp*_006 followed by rebound, and only modest effects of purified RBP alone (Fig. 5C). In contrast, a two-phage cocktail (RCIP0011 plus RCIP0011Δ*rbp*_016∷*rbp*_006) produced the strongest and most sustained inhibition, maintaining a substantially lower OD_600_ throughout the assay and a markedly reduced terminal density. This enhanced effect likely reflects functional complementarity between the two phages, as their distinct receptor specificities may enable broader targeting of bacterial cells with different surface-antigen states and thereby reduce the likelihood of escape. Consistently, suppression index analysis showed low values for single-phage treatments (10%–20%) but a much higher value for the cocktail (approximately 70%), significantly exceeding either phage alone (Fig. 5D). Together, these results indicate functional complementarity of phages with distinct receptor specificities and support an RBP-modularity framework in which diversifying receptor usage raises the barrier to bacterial escape under fluctuating surface-antigen states.

## DISCUSSION

*In vitro* experiments show that capsule inactivation—often *via* IS insertions or mutations in the *cps* cluster—is common under nutrient-rich conditions but rare under nutrient-poor conditions (28). *In vivo*, capsule loss or downregulation repeatedly emerges during gut or urinary-tract colonization (29, 30), where capsule-deficient derivatives can outcompete encapsulated ancestors in specific niches but display reduced dissemination and invasive potential. Phage-evolution experiments further indicate that resistance frequently arises through capsule-biosynthesis mutations: these protect against capsule-targeting phages but impose costs in serum resistance, virulence, and *in vivo* fitness. Together, these observations suggest that capsule-deficient lineages are likely widespread in natural *K. pneumoniae* populations, and phages that exploit capsule-loss variants or alternative surface structures may be selected in these settings. However, systematic analyses of *Klebsiella* phages that recognize non-capsular receptors remain limited. Here, we show that *Webervirus* phages segregate into two functional groups: capsule-dependent phages that display KL-specific lysis on encapsulated strains but largely lose activity on matched capsule-deficient derivatives, and capsule-inhibited phages that are inactive on encapsulated backgrounds yet become broadly lytic once the capsule is removed. We further identify the O-antigen as the receptor for this second group, indicating that *Webervirus* phages can exploit both capsular and non-capsular receptors to track capsule diversification and loss. However, this CPS-versus-OPS framework should not be assumed to apply uniformly across all *Klebsiella*-targeting phages. Different genera likely use distinct tail architectures and receptor-recognition strategies, which may lead to different receptor-use patterns. Consistent with this, our previous work on *Przondovirus* phages also linked host specificity to RBP type and capsule serotype, but with a different pattern (31). We therefore view the present framework as *Webervirus*-associated rather than universal.

Given that RBPs govern host recognition, we focused on RBP diversity. Prior work has largely emphasized capsule depolymerases and capsule-defined tropism (32, 33), whereas RBPs targeting non-capsular receptors are comparatively undercharacterized and often described in isolated cases. Where receptor switching has been examined, it is typically inferred from laboratory-derived host-range mutants rather than from systematic surveys of naturally occurring RBP diversity. In our data set, *Webervirus* RBP phylogeny resolves a clear division between O-antigen-targeting and CPS-targeting types, which is supported by functional assays. Extending the analysis to additional *Webervirus* RBPs retrieved from the NMDC phage portal (Global Catalogue of Phage) reproduced the same stratified structure (Fig. S10), with many sequences clustering with our experimentally validated OPS-targeting RBPs, suggesting that OPS targeting may be common within *Webervirus*. This is consistent with an ecological view in which the capsule is not constitutively expressed: when the capsule is downregulated or lost under nutritional, anatomical, or phage pressures, the O-antigen becomes more exposed and OPS-targeting phages are poised to exploit these capsule-poor states.

Consistent with prior work, capsule-targeting RBPs show narrow KL tropism, with RBPs recognizing the same capsule type clustering together (34); likewise, our CPS-targeting (K) RBPs partition into multiple KL-associated clades. In contrast, OPS-targeting RBPs form a single coherent clade despite spanning distinct O-type specificities, ranging from single-O recognition to broader coverage across two, three, or four O-antigens. This difference may reflect the tighter constraints on capsule-depolymerizing RBPs, which must couple recognition with enzymatic depolymerase activity, whereas OPS-RBP interactions may be more permissive (35, 36). Within the OPS-targeting clade, nonsynonymous substitutions on a shared structural scaffold likely fine-tune binding, contributing to variation in O-type breadth.

Finally, the combination of a conserved morphogenesis backbone and localized divergence at the RBP locus is consistent with recombination-driven replacement of host-recognition genes in tailed phages. Supporting this, experimentally swapping RBPs between *Webervirus* phages was sufficient to reprogram host preference, switching recognition from O-antigen to capsule. These findings support RBP turnover as a mechanism for adaptation to shifts in *K. pneumoniae* surface architecture and provide a rationale for cocktail design: a two-phage cocktail combining RCIP0011 and RCIP0011Δ*rbp*_016∷*rbp*_006 suppressed Kp067 growth by about 70% and outperformed either phage alone in growth-curve assays, consistent with complementary targeting of capsule-poor versus encapsulated receptor states. Together, our results position *Webervirus* as a tractable model for modular RBP-mediated receptor switching between capsule and O-antigen and provide a mechanism-based framework for receptor-informed phage classification and rational cocktail design against heterogeneous, capsule-variable *K. pneumoniae* populations.

## MATERIALS AND METHODS

### Bacterial strains and culture conditions

All bacterial strains, phages, and plasmids used in this study are provided in Table S6. *K. pneumoniae* and *Escherichia coli* strains were grown at 37°C in LB medium (10 g/L tryptone, 5 g/L yeast extract, and 10 g/L NaCl) with shaking at 200 rpm unless otherwise indicated. Antibiotics were added when appropriate as follows: kanamycin (50 µg/mL; *E. coli* and *K. pneumoniae*), gentamicin (20 µg/mL; *E. coli* and *K. pneumoniae*), apramycin (100 µg/mL; *E. coli* and *K. pneumoniae*), and tigecycline (4 µg/mL for *E. coli*; 8 µg/mL for *K. pneumoniae*).

### Phage propagation

*Klebsiella* phages were recovered by the double-layer agar overlay plaque assay as described previously (37). All phages were plaque-purified three times prior to use. For phage propagation, multiple plaques from soft-agar plates after overnight incubation were scraped into 5 mL of SM buffer and incubated at room temperature for at least 3 h. The resulting suspension was collected, treated with chloroform to lyse residual bacteria, and filtered through a 0.22 μm filter. Phage titers were determined by the double-layer agar method.

### Transmission electron microscopy

Bacterial cells were negatively stained with uranyl acetate and examined by TEM. Images were acquired on a JEM-1400 TEM (JEOL) operated at 80 kV to visualize the capsular halo.

### Uronic acid assay

CPS was extracted as previously described (29) and quantified by a uronic acid assay. Overnight LB cultures were adjusted to OD_600_ = 1.0 and treated with 1% Zwittergent 3-14 in 100 mM citric acid (50°C, 20 min). After centrifugation (15,000 × *g*, 5 min), CPS in the supernatant was precipitated with ethanol (4°C, 20 min), pelleted, and resuspended in water. Uronic acids were measured by mixing samples with sodium tetraborate in sulfuric acid (100°C, 5 min), followed by the addition of 3-phenylphenol in NaOH and reading A_520_. Glucuronic acid equivalents were calculated from a glucuronolactone standard curve and reported as micrograms per OD_600_ unit. Fresh LB was used as the blank, and each sample was assayed in triplicate.

### Host-range determination

Phage lytic activity was tested against *K. pneumoniae* isolates (*n* = 51) by spot assays. Briefly, bacterial lawns were prepared on LB agar and challenged with 3 µL of phage lysate (1 × 10^9^ PFU/mL), followed by incubation at 37°C for 16–18 h. All assays were performed in triplicate. Outcomes were recorded as lysed (bright, clear lysis zone) or non-lysed (no visible clearing). To validate productive infection beyond qualitative spot assay results, a subset of representative phages was selected for plaque assay on selected hosts (9, 38). Serially diluted phage suspensions were plated using the double-layer agar method, and plaque-forming units were enumerated after overnight incubation at 37°C to determine phage titer (PFU/mL). For the focused panel comprising four representative strains and their capsule-deficient and O-antigen-deficient derivatives tested against 18 phages, phage susceptibility was first screened by spot assay and then quantified by plaque assay-based titer determination. EOP was calculated as the ratio of phage titer on the test strain to that on the corresponding reference strain (39). For O-antigen-deficient derivatives, the matched capsule-deficient parental host served as the reference. Titers below 100 PFU/mL were considered below the limit of detection and recorded as ND.

### Phage genome extraction, sequencing, and bioinformatics analysis

Phage genomic DNA was extracted from high-titer lysates (≥1 × 10^9^ PFU/mL) using the Lambda Phage Genomic DNA Kit (ZOMANBIO, Beijing, China) according to the manufacturer’s protocol. Libraries were sequenced on an Illumina NovaSeq 6000 platform (PE150; Novogene). Reads were assembled with SPAdes v.3.15.5 (40). ORFs were predicted and annotated with Prokka v.1.14.6 (41) using --kingdom Viruses and --metagenome. Protein functions were assigned by (i) NCBI Batch CD-Search with Pfam and TIGRFAM enabled (42), (ii) KofamKOALA for KEGG ortholog assignment (43), and (iii) BLASTp against VOGDB (https://vogdb.org/) using cutoffs of >40% identity and >40% alignment coverage.

### Identification of TerLs and RBPs

TerL proteins were identified from Prokka annotations and, when necessary, confirmed by HHpred (44). RBPs (tail fiber/tailspike-like proteins) were retrieved from annotations when available; for genomes lacking explicit RBP calls, candidate loci were nominated by comparative genome alignment in Easyfig and refined with HHpred to support remote homology and adjust gene boundaries. RBP structures were predicted with AlphaFold3 (45) using full-length amino acid sequences and visualized in PyMOL (v.2.5.8).

### Phylogenetic analysis of TerLs and RBPs

TerL and RBP amino acid sequences were aligned using MAFFT (46) and trimmed with trimAl (47). Maximum-likelihood phylogenetic trees were inferred using IQ-TREE (48) with automatic model selection, and branch support was estimated by bootstrap resampling (parameters as specified in the analysis script). Trees were visualized and annotated in iTOL (49).

### Screening and genomic analysis of phage-resistant mutants

RCIP0022-resistant derivatives of *K. pneumoniae* KpRJF293cap^-^ were selected by infecting cultures with RCIP0022 (MOI = 10, 37°C) and isolating single colonies on LB agar. Resistant clones were purified and confirmed by double-layer agar assays. Genomic DNA from three independent clones (KpRJF293cap^-^-M1, -M2, -M3) was extracted (TIANGEN) and sequenced using Nanopore PromethION and Illumina NovaSeq 6000 (PE150; Novogene). Complete genomes were assembled from Nanopore reads with Flye, polished with Illumina reads using NextPolish (50), and aligned to the parental KpRJF293cap^-^ genome with progressiveMauve (51). Variants were called using an in-house Perl script and verified by read mapping to the parental genome and inspection of local coverage.

### Construction of gene knockout mutants and complemented strains

Deletion of *wbbN* (in the O1 strain KpRJF293cap^-^ and the O2 strain Kp8-181cap^-^) and *wbdD* (in the O3 strain Kp4-147cap^-^) was generated using a CRISPR-Cas9/λRed recombineering system based on pCOLADRed and pCas9gRNA (Table S8). Strains carrying pCOLADRed were co-transformed with pCas9gRNA and a PCR-derived repair template containing left/right homology arms flanking *wbbN* or *wbdD*. Transformants were selected on antibiotic LB agar, and mutants were verified by colony PCR and Sanger sequencing (Table S5).

Deletion of *wbbB* in *K. pneumoniae* Kp1189cap^-^ was generated using a temperature-sensitive CRISPR-Cas9 system based on pCasKP-apr and pSGKP-tetX4, as described previously (52). The pCasKP-apr strain was transformed with pSGKP-tetX4 and a PCR-derived repair template flanking *wbbB*, selected on antibiotic LB agar, and confirmed by colony PCR and Sanger sequencing (Table S5).

For complementation, aTc-inducible vectors pGm and pApr (P15A ori; Gm^R^ and Apr^R^) were derived from pdCas9-gRNA by deleting dCas9 while retaining the aTc-inducible expression cassette. *wbbN* and *wbdD* were cloned into pGm, and *wbbB* into pApr. All constructs were verified (Table S5) and introduced into the corresponding deletion mutants for complementation assays.

### LPS extraction and electrophoresis

LPS was extracted using an LPS extraction kit (iNtRON Biotechnology) following the manufacturer’s instructions. Samples were resolved on 15% SDS-PAGE gels and silver-stained (Solarbio).

### Cloning, expression, and purification of phage RBPs

Candidate RBP genes were PCR-amplified from phage genomic DNA and Gibson-assembled into pET-28a(+) linearized with *Nde*I/*Xho*I. An EGFP control plasmid in the same backbone was generated by PCR-linearizing pET-28a(+) and inserting the EGFP coding sequence using the same strategy. Constructs were propagated and sequence-verified in *E. coli* TOP10, then transformed into *E. coli* BL21(DE3) for expression.

BL21(DE3) transformants were grown overnight in LB-kanamycin (50 µg/mL) at 37°C (220 rpm), diluted 1:100, and cultured to OD_600_ = 0.6–0.8. Expression was induced with 0.5 mM IPTG at 18°C overnight (150 rpm). Cells were harvested (5,000 × *g*, 10 min, 4°C), washed with PBS, and resuspended in lysis buffer (50 mM Tris-HCl, 200 mM NaCl, pH 8.0). Lysates were sonicated, clarified (10,000 × *g*, 1 h, 4°C), and the supernatant was purified on Ni-NTA resin. Eluted His-tagged proteins were assessed by SDS-PAGE, and high-purity fractions were concentrated using 30 kDa MWCO centrifugal filters. Primer sequences are listed in Table S6.

### RBP depolymerase activity (spot assay)

Purified RBPs were assessed for capsule depolymerase activity using a spot assay on bacterial lawns. *K. pneumoniae* cultures (KL1, KL2 and KL57) at an OD_600_ of 0.6–0.8, together with their corresponding capsule-deficient derivatives, were mixed 1:100 (vol/vol) with LB soft agar (0.6%) and overlaid onto LB agar plates. Purified RBPs were serially diluted in PBS (500, 50 and 5 ng per 4 μL), and 4 μL of each dilution was spotted onto air-dried lawns; matched phage stocks were spotted in parallel. Plates were incubated at 37°C for 12 h, and halo formation was recorded.

### Alcian blue staining

CPS depolymerization was assessed by incubating bacteria at an OD_600_ of 0.6–0.8 with purified capsular depolymerase at 37°C for the indicated time, followed by CPS extraction and Alcian blue staining. CPS was extracted using a commercial kit (Solarbio) per the manufacturer’s instructions and resolved on 6% SDS-PAGE. Gels were washed in fix/wash solution (25% ethanol, 10% acetic acid; 50°C) three times (5, 10 and 15 min), stained with 0.125% Alcian blue in the same solution (15 min, 50°C), and destained with fix/wash solution. CPS was visualized as Alcian blue-positive material (53).

### Bacterial cell growth assay with recombinant RBPs

*K. pneumoniae* cultures grown to an OD_600_ of 0.6–0.8 were adjusted with fresh LB to an OD_600_ of 0.3 (approximately 10⁷ CFU/mL) and then diluted 1:1,000 (vol/vol) in LB. Purified RBP proteins (0.1 mg/mL stock; 50 μL per well, with 10-fold serial dilutions where indicated) were mixed with the bacterial suspensions and preincubated at 4°C for 30 min to allow receptor binding. Phage stocks were then added to achieve a final MOI of 1. Control wells contained bacteria alone and bacteria plus phage. All experiments were conducted in triplicate. Bacterial growth was continuously monitored by measuring the OD_600_ using a Bioscreen C automated growth analyzer (Growth Curves Ltd., Helsinki, Finland) at 37°C for up to 24 h, as described previously (54).

### Fluorescence and confocal analysis of GFP-RBP binding

Overnight *K. pneumoniae* cultures were normalized to OD_600_ = 1.0. Aliquots (500 µL) were pelleted (5,000 × *g*, 4°C, 4 min) and resuspended in 120 µL PBS-T (PBS, pH 7.4, 0.1% Tween-20). GFP-RBP was added and incubated for 30 min at 4°C with rotation. Cells were washed twice with 1 mL PBS-T and resuspended in 200 µL PBS-T. Fluorescence was measured by transferring 150 µL to a black 96-well plate and reading on a FLUOstar Omega microplate reader (Ex 485 nm/Em 520 nm). For confocal microscopy, 5 µL of the same suspension was applied to a confocal dish and imaged on a Leica SP8 confocal microscope equipped with a 100× oil-immersion objective, as described previously (55).

### Phage engineering

Phage engineering was performed as described previously (34). A donor cassette carrying the desired RBP gene and flanking homology arms was assembled into the pCOLADRed backbone by Gibson assembly to obtain pCOLADRed-donor plasmid. This plasmid was introduced into the RCIP0011 phage propagation host by electroporation. Transformants were selected on kanamycin-containing plates and cultured in kanamycin-containing medium supplemented with 1% (wt/vol) L-arabinose to induce the λ Red recombination system. When cultures reached an OD_600_ of 0.5, wild-type phage RCIP0011 was added and incubated for 3 h at 37°C with shaking. Candidate recombinants were isolated by plaque purification on a selective host using a double-layer agar assay and verified by diagnostic PCR. Confirmed recombinant phages were passaged three times on the new host to ensure stability. Primers are listed in Table S7.

### Engineered phage cocktail and suppression index

Engineered phage-mediated growth inhibition of *K. pneumoniae* Kp067 was quantified using a Bioscreen C automated growth analyzer. Briefly, log-phase Kp067 cultures were adjusted to an OD_600_ of 0.3 (approximately 10⁷ CFU/mL), and 100 μL was dispensed per well into honeycomb plates. Phage suspensions were added to achieve a final MOI of 1.0 (single phage, 100 μL; two-phage cocktails, 50 μL each). Wells receiving an equal volume of LB instead of phage served as no-phage controls. Plates were incubated with continuous shaking, and OD_600_ was recorded hourly for 24 h. Each condition was tested in three independent experiments.

Growth curves were generated from OD_600_ readings, and the area under the curve from 0 to 24 h (AUC_0–24h_) was calculated in GraphPad Prism 10.5.0 (GraphPad Software). The suppression index was defined as (AUC_no phage − AUC_phage) / AUC_no phage.

### Statistical analysis

Statistical analyses were performed in GraphPad Prism 10.5.0 (GraphPad Software). Two-group comparisons used two-tailed Student’s *t*-tests (paired for matched host pairs; unpaired otherwise). Comparisons among three or more groups used one-way ANOVA with Tukey’s multiple comparisons test. Data are presented as mean ± SD; *n* denotes independent experiments. *P* < 0.05 was considered statistically significant.

## Data Availability

The *K. pneumoniae* strains and phages used in this study have been deposited in the National Center for Biotechnology Information (NCBI; https://www.ncbi.nlm.nih.gov/) under the following BioProject PRJNA1395354: SAMN54360374 (KpRJF293cap^−^), SAMN54360375 (KpRJF293cap^−^M1), SAMN54360376 (KpRJF293cap^−^M2), and SAMN54360377 (KpRJF293cap^−^M3). The other corresponding metadata for the bacterial isolates used in this study, including KL type, O type, ST type, genome accession number, and isolation source, are summarized in Table S1. The NCBI accession numbers for the 47 phage genomes analyzed in this study are provided in Table S2.

## SUPPLEMENTAL MATERIAL

Supplemental Figures mbio.00046-26-s0001.docx Fig. S1-S10.

Supplemental Tables mbio.00046-26-s0002.xlsx Tables S1-S8.

ASM does not own the copyrights to Supplemental Material that may be linked to, or accessed through, an article. The authors have granted ASM a non-exclusive, world-wide license to publish the Supplemental Material files. Please contact the corresponding author directly for reuse.
