# Supplementary material for: Webervirus phages exploit capsule- and O-antigen-targeting receptor-binding proteins to access diverse Klebsiella pneumoniae hosts
Source: mBio. 2026 Jun 22;17(8):e00046-26. doi: 10.1128/mbio.00046-26 (PMC13463945; doi:10.1128/mbio.00046-26)
Supplement: Supplementary file 1 [file mbio.00046-26-s0001.docx]

**Legends of supplementary materials**

**Supplementary Fig 1.** ***Webervirus* is the most abundant genus of *Klebsiella* phages**

Pie chart showing the proportions of *Klebsiella* phages across different genera retrieved from the NCBI Virus database (accessed 24 December 2025; n = 1,392). All complete *Klebsiella* phage nucleotide sequences were retrieved from the NCBI Virus portal (accessed 24 December 2025).

**Supplementary Fig 2. Phylogenetic tree of 51 clinical *K. pneumoniae* strains**

Genomes (n = 51) were annotated with Prokka (v1.14.6), and a core-gene alignment was generated with Roary (v3.13.0). A maximum-likelihood phylogeny was inferred from the core-gene alignment using RAxML (v8.2.12) under the GTRGAMMA model. phylogenetic tree of the clinical *K. pneumoniae* isolates used in this study. Outer rings indicate KL type, O type, and MLST lineage (ST23, ST11, and other STs) as shown. Outer symbols denote carriage of carbapenemase genes: red stars, *bla*_KPC_; blue stars, *bla*_NDM_; purple stars, *bla*_KPC_ + *bla*_NDM_; green stars, *bla*_IMP_. Bootstrap support values exceeding 90% are demonstrated at the corresponding nodes.

**Supplementary Fig 3. Phenotypic differences between encapsulated and capsule-deficient *K. pneumoniae* strains.**

A. Colony morphology encapsulated strain (Kp8-181cap^+^; opaque) and its capsule-deficient derivative (Kp8-181cap^-^; translucent) on LB agar. B. TEM images of Kp8-181cap^+^ and its capsule-deficient derivative (Kp8-181cap^-^), showing a prominent capsular layer surrounding the parental cell but no detectable capsule in the cap^-^ variant (scale bars, 1 μm). C. Quantification of capsular polysaccharide by uronic acid assay in the indicated strain backgrounds, comparing encapsulated (cap^+^) and capsule-deficient (cap^-^) derivative. Points represent independent measurements; bars summarize the data with error bars. *P* < 0.01 (**), *P* < 0.001 (***). Statistical test is specified in Methods.

**Supplementary Fig 4.** **AlphaFold3 structural predictions for three RBPs**

InterPro sequence-based annotation did not identify pectin/pectate lyase-like domains in RCIP0077_RBP, RCIP0194_RBP, or RCIP0195_RBP. However, AlphaFold3 structure predictions were consistent with a β-strand–rich fold characteristic of polysaccharide depolymerase-like fold in all three proteins. RCIP0077_RBP (green), RCIP0194_RBP (blue), and RCIP0195_RBP (pink).

**Supplementary Fig 5.** **SDS-PAGE analysis of soluble RBP and RBP-GFP fusions.**

SDS-PAGE of purified RBPs and corresponding GFP fusion proteins. A. Purified RBPs. Lane 1: molecular weight markers, Lane 2: Purified RCIP0025_RBP, Lane 3: Purified RCIP0135_RBP, Lane 4: Purified RCIP0207_RBP; B. Purified Cluster I RBPs. Lane 1: molecular weight markers, Lane 2: Purified RCIP1866_RBP, Lane 3: Purified RCIP0005_RBP, Lane 4: Purified RCIP0022_RBP. C. Purified RBP-GFP fusions. Lane 1: molecular weight markers, Lane 2: RCIP1866_RBP-GFP, Lane 3: RCIP0005_RBP-GFP, Lane 4: RCIP0022_RBP-GFP.

**Supplementary Fig 6. Functional assays of CPS-depolymerizing RBPs.**

A. Activity of purified RBPs and phage stocks on *K. pneumoniae* lawns. Spot tests were performed by applying purified RBPs to bacterial lawns of encapsulated strains and their corresponding capsule-deficient derivatives. B. SDS-PAGE analysis of purified CPS incubated with selected purified RBPs. CPS degradation was detected after incubation and visualized by Alcian blue staining. Molecular-mass standards (kDa) are indicated.

**Supplementary Fig 7. O-locus disruption in RCIP0022-resistant mutants and representative structures of major *K. pneumoniae* O antigens.**

A. Schematic of the O-antigen biosynthesis locus in the capsule-deficient derivative KpRJF293cap^-^ and three independent RCIP0022-resistant mutants isolated by serial passage. Resistance-associated mutations include IS*5* insertions and a premature stop codon in *wzt* (locus tag 44129) (mutation sites indicated in red). B. Pie chart showing O-antigen type frequencies among 2,756 complete *K. pneumoniae* genomes in NCBI RefSeq (accessed March 2025). The four dominant O types are O2 (37.8%), O1 (32.7%), O3 (12.4%) and O13 (7.9%). O2 aggregates O2a/O2afg/O2ac, and O3 aggregates O3a/O3b. C. Schematic of the *K. pneumoniae* surface envelope highlighting capsule and LPS (O antigen and core LPS/OS, and lipid A) in the outer membrane (OM) and inner membrane (IM), with representative repeat-unit structures of major O antigens(O2/O1/O3/O13) shown at right. Sugar symbols: Galp, galactopyranose; Galf, galactofuranose; Man, mannose; Rha, rhamnose; Glc, glucose.

**Supplementary Fig 8. Silver-stained SDS–PAGE profiles of LPS from four *K. pneumoniae* strains and matched capsule- and O-antigen-deficient derivatives.**

LPS preparations from the indicated *K. pneumoniae* strains were separated by SDS-PAGE and visualized by silver staining. For each background (KpRJF293, Kp8-181, Kp4-147, and Kp1189), lanes show the encapsulated strain, the capsule-deficient derivative (cap^-^), and the capsule- and O-antigen-deficient derivative (cap^-^Δ*wbbN*, *wbdD* or *wbbB,* as indicated), with a molecular-weight marker at left. Brackets denote the regions corresponding to the long O-antigen ladder and to core oligosaccharide plus lipid A (core-OS + lipid A).

**Supplementary Fig 9** **Genomic Comparison and TerL Phylogeny of *Webervirus* phages.**

A. Comparative genomic analysis of the representative *Webervirus* phages. Genome organization of each phage is shown. Arrows indicate coding sequences and are colored by predicted function, as follow: DNA (light blue), morphogenesis (yellow), hypothetical proteins (white), capsule-associated RBP (K_RBP; pink), O-antigen-associated RBP (O_RBP; orange), DNA modification (green), and nuclease (purple). Grey shading represents >70% nucleotide identity. B. Maximum-likelihood phylogeny of *Webervirus* TerL. Tip labels represent individual phage isolates. Colored bars annotate the receptor-binding protein (RBP) clusters (I–V) and receptor classes (K, capsule; O, O-antigen).

**Supplementary Fig 10. *Webervirus* RBPs phylogeny reveals receptor-linked clustering.**

*Webervirus* phage genomes used for the expanded analysis were downloaded from the Global Catalogue of Phage database (NMDC phage portal; dataset *Phage.17770sequence.fasta.gz*). RBPs were identified by using our curated set of 47 RBPs as query sequences to search for homologous RBPs in the downloaded *Webervirus* genomes. Homologous RBP sequences were aligned, and an amino-acid maximum-likelihood phylogeny was inferred following the workflow described above. Tip labels denote individual RBP sequences. Colored bars annotate receptor class: O-antigen-targeting RBPs form a single coherent cluster, whereas capsule-associated RBPs further segregate by capsular KL type, with KL1-, KL2-, and KL57-associated RBPs forming distinct clusters. Gray shading denotes RBPs whose receptor class could not be assigned based on available evidence. Bootstrap support values >90% are shown at the corresponding nodes.
